# Hydrogels in Hand Sanitizers

**DOI:** 10.3390/ma14071577

**Published:** 2021-03-24

**Authors:** Carla Villa, Eleonora Russo

**Affiliations:** Section of Medicinal and Cosmetic Chemistry, Department of Pharmacy, University of Genova, Viale Benedetto XV, 3-16132 Genova, Italy

**Keywords:** hand rub sanitizers, hydrogel, disinfectants

## Abstract

Hand hygiene can be considered a strategic key useful in the containment of infections such as COVID-19 both at home and in communities because it can dramatically reduce the widespread outbreak of infections. In case of the unavailability of soap and water, “instant” hand sanitizers are recommended because their application can be considered easy, versatile, quick and often less aggressive for the skin. For these reasons, alcoholic and alcohol-free hand rub gels can be considered the best performing formulations on the market. Together with disinfectants and antiseptic agents, hydrogels play a fundamental role in obtaining stable formulations and are easy to disperse, with a pleasant skin feel and an overall good performance. Several compounds commonly used in the pharmaceutical, cosmetic and food industry are available for this purpose, in particular, cellulose derivatives and synthetic polymers derivatives. Each of them is available in several grades, presenting different thickening behavior, rheological properties and compatibility with other ingredients, alcohols in particular. For all these reasons, it is important to explore hydrogel properties and behaviors in different contexts (i.e., hydroalcoholic and aqueous media) in order to develop new and performing hand rub gels, always taking into account the different international legal frameworks regarding disinfectant and sanitizing formulations.

## 1. Introduction

It has been estimated that there are not less than 10,000 organisms per cm^2^ of normal skin, pathogenic transient flora included [1], and hands are regarded as one of the principal sites responsible for transmitting infections, such as pandemic ones [2,3,4]. Therefore, hand hygiene and disinfection can be considered strategic keys in the containment of several infections, such as COVID-19, both at home and in communities because they can dramatically reduce the widespread outbreak of pathogens and they can also prevent the transmission of them to food [5]. Hand sanitization includes (1) handwashing, in particular using a common soap in the presence of water; (2) handwashing, using a detergent (possibly antiseptic ones) with water; and (3) hand sanitization using alcoholic hand rubs [6].

The Centers for Disease Control and Prevention (CDC) recommends washing hands with soap and water for at least 20 s. Rinse-off detergents are considered better performing than hand rub sanitizers in the removal of certain pathogens such as *Norovirus*, *Cryptosporidium,* and *Clostridioides difficile* [7,8], but when they are not available, or when repeated hand washing alters the skin’s natural barrier [9], “instant” hand sanitizers are recommended [10]. The main goal of these topic sanitizers (antiseptic handrub or handrub products) is to remove or reduce the level of transient bacteria and viruses. In particular, an “instant” hand sanitizer is intended to be applied to dry hands, rubbed thoroughly over the fingers and hand surfaces for at least 30 s, and completely air-dried. They are formulated as foam, gel, or liquid preparations [11,12,13] and they can be classified as alcohol-based rubs (ABR) or alcohol-free rubs (AFR), according to the active, antiseptic ingredients used. Their application can be considered more versatile, convenient, quick, and less irritating [14,15] when compared with the use of rinse-off detergents. ABRs generally contain alcohol, water, and other ingredients (in particular humectants and emollients); hands are their target to quickly destroy microorganisms and suppress their growth, in a broad germicidal spectrum. Nevertheless, their effect on pathogens seems short-lived and they do not have a strong activity against protozoa, bacterial spores, and some non-enveloped viruses [4].

The “WHO Guidelines on Hand Hygiene in Health Care: First Global Patient Safety Challenge Clean Care Is Safer Care” provides a useful scientific review on hand hygiene argument and suggests the best procedures in health care. The WHO describes a sanitizing hand rub as: “An alcohol-containing preparation (liquid, gel or foam) designed for application to the hands to inactivate microorganisms and/or temporarily suppress their growth. Such preparations may contain one or more types of alcohol, other active ingredients with excipients, and humectants” [16]. The Centers for Disease Control and Prevention (CDC) and the World Health Organization recommend the use of ABR formulations containing 60 to 95% alcohol as the best practice for hand sanitization, but only when hands are not noticeably dirty [17]. ABRs are considered a better performing tool in minimizing hand contamination, especially when compared to soap and water [14]; however, the activity against non-enveloped viruses is still debated [18,19,20,21,22,23,24], particularly for formulations containing < 75% alcohol [25,26,27]. Moreover, only a few researches refer to skin toxicity due to high alcohol content [28]. To help countries in the adoption of alcoholic hand rubs as the best practice for hand hygiene and sanitization, the WHO has identified two simple formulations for local preparation, when commercial products may be unavailable [29]. These formulations (Table 1) are reported in the “Guide to Local Production: WHO-recommended Handrub Formulations”. The choice of the selected ingredients is due to three main factors: low cost, availability, and microbicidal activity [21]. These formulations are recommended for local production, recommending a maximum of 50 L per lot in order to ensure safe production and storage.

The denatured alcohol works as the topical antiseptic or antimicrobial agent; hydrogen peroxide is included to inactivate contaminating bacterial spores in the final solution, but it cannot be considered an antiseptic ingredient. Glycerin is useful as the humectant agent; it affects the viscosity of the final product and provides a minimal level of moisturization to the skin, but an excessive amount of glycerin can reduce the germicidal activity of isopropanol and ethanol, as cited by a footnote of the World Health Organization (glycerin mixed with alcohols forms an azeotrope that can affect their activity. As an alternative, PEG-10 dimethicone and PEG-7 glyceryl cocoate can be use as refatting agents [30]). Water performs as a solvent and vehicle to help deliver the final product to the skin. The addition of perfumes or dyes is not recommended.

Alcohol-free products (AFR) contain chemicals (biocides) with antiseptic properties, often used at low concentrations, and can be considered relatively safer than ABR, especially for children, also being non-flammable [31,32,33]. However, they are less preferred by the health organizations [34,35] for fighting COVID-19 because of their lower efficacy and because they are not broad-spectrum agents [36]. Their antimicrobial action can be affected by different variables, such as other ingredients in the formulation components, dilution, the presence of an organic load, etc.

These liquid formulations present some difficulties to handle, potentially leading to the delivery of insufficient doses of active agents on the hands and to an overall reduction in hygiene compliance [37,38,39]. In a recent study on ABRs [40], researchers investigated how many elements such as skin health, education, and user acceptance of ABRs might affect healthcare workers’ hand sanitization during and after application. The results show that despite the benefits that liquid products give (clean sensation, smooth and moisturized feel), the difficult handling and applying of the products cancel out the advantages of such formulations. Even if the WHO has recommended and described the preparation of two liquid hydro-alcoholic hand rub formulations, in the consumer market, hydrogel sanitizers are becoming increasingly popular. In fact, viscosity plays a significant role in many key aspects of a hand sanitizer gel’s functionality. Efficiency, performance, and customer perception are closely linked to viscosity values. The literature reports only a few papers that highlight the role of hydrogels in hand disinfection, but these semi-solid preparations present numerous advantages over liquid forms, not only for their ability to disinfect, but also for the ease with which they can be dispensed and used on-the-go. Hydrogels can be considered more desirable than liquid forms thanks to fast absorption and drying, a pleasant hand feel, absence of stickiness, mild smell, and clean and cold sensation during application. Coldness can also help in monitoring the complete hand covering. Hydrogels, when compared to liquid-based preparations, are easier to have at hand and more practical to deliver on-the-spot, because of their simplicity of delivery and low risk of leakage. Moreover, they can reduce the alcoholic evaporation rate, allowing a better spreadability and a deeper penetration through contaminating organisms. On the other side, they can present negative features such as skin dehydration after prologued use and a stinging sensation for contact to broken skin. As regards adverse reactions, the most commonly reported ones are allergic and irritant contact dermatitis [36]. The main problem regards the depletion of the skin lipophilic defense, in particular after a repeated and prolonged exposure to fat-dissolving alcohols [41,42]. In a study carried out on a selected group of nurses, the compliance of a number of sanitizing formulations was investigated. It emerged that all the nurses chose liquids as the least favorite format, mainly for the difficulties in application, for the low covering, low doses, and unpleasant, uncontrolled dripping. Liquid bowls were also more difficult to handle than gel and foam dispensers [16,40].

Taking into account all these statements, the aim of this review is to highlight the properties and advantages of hydrogels in regard to hand sanitizers, with particular attention to alcohol-based hydrogels that can be considered the best performing and most active topic infection preventive tools [43]; having different compositions, sanitizing hydrogels need a deep study for their correct formulation together with an appropriate labelling, dispenser, and closure so as to achieve a proper dose/amount of the sanitizer for an efficient disinfection on each use [44]. For a better comprehension of all these concepts, the review will deal with different aspects related to the sanitizing approach such as the main biological differences between bacteria and viruses, the principal ingredients and products useful for their deactivation, the most important properties and characterizations of hydrogels, more information regarding carbomers and cellulose derivatives, and a brief overview on the current international regulation.

## 2. Bacteria vs. Viruses

**Figure 1:**
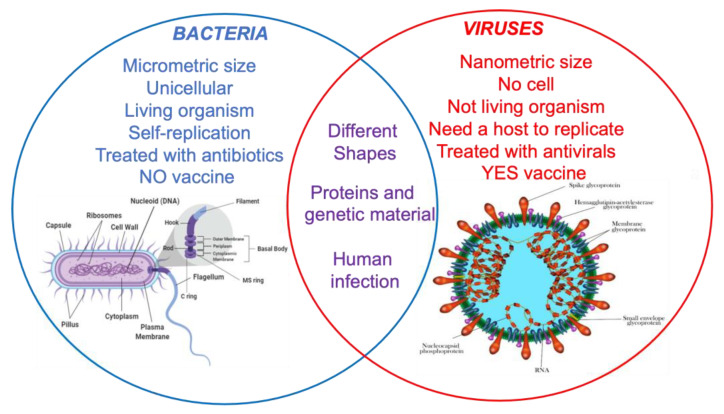
Main differences and similarities between bacteria and viruses [45,46].

Viruses and bacteria are microorganisms that exploit all the environments where different life forms are present. The main differences concern morphology, size, and replication capacity (Figure 1 [45]). Bacteria are unicellular microorganisms called prokaryotes that can grow in very different environments, including harsh conditions such as acidic hot springs and radioactive waste, where they form dense aggregations on surfaces called biofilms. They are autonomous for reproduction, and they can be grown on synthetic soils. The bacterial cell is a complex structure, with an inner cell membrane and an outer cell wall, which ranks the bacteria into Gram-positive and Gram-negative that can have an enclosing capsule of polysaccharides for extra protection. The membrane coats a cytoplasm containing nutrients, proteins, DNA, and other essential components of the cell. A single, circular chromosome which carries the bacterium’s genetic information is found in the nucleoid together with ribosomes for protein synthesis. The cell can be coated by a rigid bacterial wall that confers a particular morphology. For this reason, bacteria with a spherical shape are called “Cocci” (i.e., *Staphylococcus, enterococcus*) and the ones with a rod shape are called “Bacilli” (i.e., *Anthrax bacillus*). The Gram-positive bacteria present an external membrane composed of peptidoglycans, while Gram-negative bacteria have an additional one, consisting of lipopolysaccharides and proteins, that covers them. On the outside, the bacterial cell can be surrounded by a capsule and equipped with appendages such as *flagella*, responsible for the cell movement, or *pili* which allow for the adhesion to host surfaces and/or tissues. As regards size, bacteria can be observed under a simple optical (light) microscope, having micrometer or sub-micrometer sized structures.

Viruses are not considered as living organisms because they do not have cells. They are relatively simple structural infectious agents consisting of genetic material, either DNA or RNA, covered by a protein “core” (Figure 1 [46]) Viruses are 10 to 20 times smaller than bacteria (of the order of magnitude of nanometers) and can be observed only by an electron microscope. Viruses are not capable of self-replicating; they need to infect cells to replicate, exploiting the host cell apparatus. There are viruses that infect plant cells, animal cells (and therefore, also human cells), and even bacterial cells; the latter viruses are called bacteriophages. Influenza, measles, and HIV are some of the best-known viruses capable of causing infections in humans.

Coronaviruses are spherical viruses and belong to the family of *Coronaviridae.* They have enveloped virions and club-shaped glycoprotein spikes in the envelope that give the viruses a crownlike, “coronal” semblance. It presents a helical or tubular nucleocapsid, equipped with a proteic shell (capsid) that contains the viral nucleic acid. The CoVs viruses belong to the genus *Beta Coronavirus*, and share similar morphology to enveloped, positive single-stranded RNA viruses [47,48]. Contrary to assumptions, being enveloped microorganisms, coronaviruses (including SARS-CoV-2) are usually less resistant than the so-called “naked” viruses, without envelopes. A disinfectant that claims virucidal action always includes efficacy against enveloped viruses, but it may not be effective against more resistant non-enveloped ones. Until they enter host cells (when they infect humans or animals) and lay on organic wet surfaces, such as human skin, viruses have few ways to defend from external attacks. Therefore, even the dryness caused by pure alcohol or the damage caused by hydrogen peroxide can be enough to destroy them. Ethanol in high concentrations is a powerful virucidal agent capable of inactivating all lipophilic viruses (vaccine, herpes, and influenza viruses) and also many non-lipophilic ones (adenovirus, rotavirus, enterovirus, but not the hepatitis virus); isopropyl alcohol is active only against lipophilic viruses; hydrogen peroxide produces free radicals that attack lipid envelopes and DNA. However, in the absence of water, protein denaturation is difficult to achieve, and for this reason, the main alcoholic products available on the market for skin disinfection (effective against viruses) are based on ethanol (73.6 to 89% *w*/*w*) or ethanol-isopropanol mixtures with an ethanolic concentration of 65% (*w*/*w*).

## 3. Handrub Sanitizers

**Figure 2:**
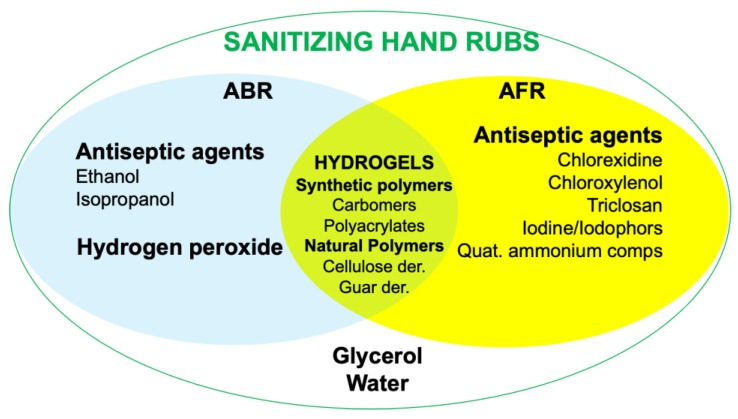
Most common antiseptic agents and excipients used in alcoholic sanitizing hand rubs (ABR) and alcohol-free sanitizing hand rubs (AFR). Hydrogels can be used to enhance the performance of both the sanitizing systems.

Sanitizing hand rubs can be classified as alcohol-based rubs (ABR) or alcohol-free rubs (AFR) according to the active antiseptic agents used. Both types share the same basic ingredients—water and glycerol—and, in the case of hydrogel preparations, also thickening agents (Figure 2). Hydrogel sanitizing hand rubs can be formulated from natural, semi-, or synthetic polymeric materials which allow an increased product performance at the hands’ skin level, carrying out a more prolonged antimicrobial activity. These gelled forms, when compared to liquid ones, ensure greater permanence on site due to their gelling properties, provide rheological control in thickening, and present a non-tacky feel, allowing the exploitation of their physical properties in performing personal care products.

### 3.1. Alcohol-Free Handrub Sanitisers

Several chemical agents are available to formulate a good sanitizing alcohol-free hand rub (Table 2); in different cases, the antiseptic agents are well-known for their antimicrobial properties and have been used from a long time, alone or in combination, for antisepsis, disinfection and preservation [49,50,51,52]. Their sanitizing activity can be influenced by different variables such as the presence of an organic load, temperature variation, different dilutions, different assays, etc. [53]. Moreover, the nature and composition of the microorganism surface can be significantly different from one cell type (or entity) to another, so the interaction efficacy of the antiseptic or disinfectant agent with the microorganism can be strongly influenced by these differences [54,55]. For these reasons, the choice of the correct compound, which acts according to its chemical functional groups (Table 2), is crucial. The most commonly used active ingredient of AFR is benzalkonium chloride, a quaternary ammonium salt, but almost all of the antiseptic compounds reported in Table 2 can be used in both liquid and hydrogel formulations at the suggested percentages, except for sodium hypochlorite. It has a long and well-developed history of use as an antiseptic and disinfecting agent, and also for dermal use (i.e, 0.05% Amukine Med^®^). However, despite its high efficiency, it is well-known that the oxidative power and alkalinity of sodium hypochlorite make this chemical compound incompatible with most of the organic compounds commonly used as excipients, in particular polymers used as gelling agents [56]. Only a few inorganic compounds can be used to obtain a useful sodium hypochlorite sanitizing gel formulation, but the complex stability leads to the use of its solution as the best choice.

### 3.2. Alcohol-Based Handrub Sanitisers

The good performance of the alcoholic hand sanitizers mainly depends on the percentage and type of the alcohol, but also on the amount applied on hands and contact time [11]. Ethanol, isopropanol or a mixture of them are the most commonly found agents in hand rubs [16]. The antimicrobial activity of alcohols is represented by their ability in protein denaturation. The most effective solutions contain 60 to 80% alcohol, resulting in lower efficiency at higher concentrations [58,59]. This paradox can be easily explained considering that proteins cannot be easily denatured without a certain amount of water [47]. To address the COVID-19 health emergency, very recently (March 2020, updated February 10, 2021), the Food and Drug Administration (FDA) published a guidance for industry on the production and distribution of hand sanitizer products for the public’s use. Here, two options about alcohol use are strongly recommended [60]:

- Ethylic alcohol not less than 94.9% (*v*/*v*) ethanol, produced by fermentation and distillation processes or by synthetic processes only if it respects USP or FCC standards (as regards impurities, such as methanol).
- Isopropyl alcohol (IPA) according to United States Pharmacopeia (USP grade).

Alcohols attacks and destroys the envelope proteins that surround some viruses, including coronaviruses, but they do not remove the carcasses of the virus from skin [61]; moreover, they are not effective against bacterial spores [62,63]. Ethanol has a higher viricidal activity than isopropanol against non-enveloped viruses [64] and it presents a better skin tolerance [65,66]. Thus, ethylic alcohol can be considered the gold standard in the alcohol choice for ABR preparations [67,68]. The United States Food and Drug Administration (US FDA), CDC, and the WHO consider concentrations from 60 to 95% (*v*/*v*) effective for disinfection, including for use against SARS-CoV-2 [16,43,60,69]; however, products with an ethanolic concentrations from 80 to 85% (*v*/*v*) showed the need of a lower contact time useful to obtain a satisfying antimicrobial activity [63,70,71]. As regards isopropanol, according to the US FDA’s Tentative Final Monograph (TFM) for health care antiseptics, the most active concentrations should range between 70 and 91.3% (*v*/*v*) [47]. In a recent study, both ethanol and isopropanol used in the WHO-recommended hand rubs have shown an efficient activity against SARS-CoV-2, [72,73].

## 4. Sanitizing Hydrogels: Properties and Characterization

**Figure 3:**
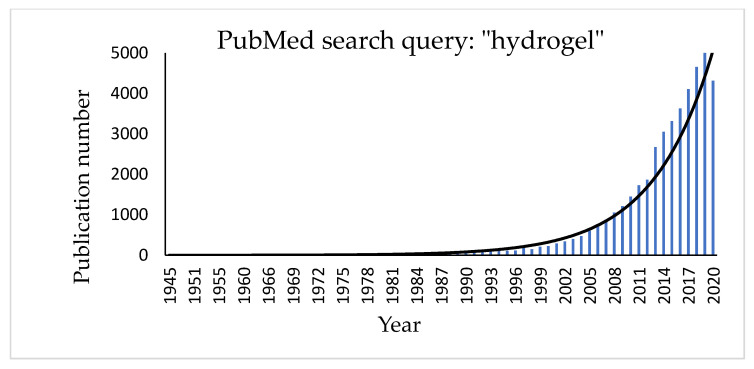
Histogram showing the increasing number of publications for the term “hydrogel” in the PubMed database.

Hydrogels are three-dimensional, hydrophilic cross-linked polymeric networks extensively swollen with water (or biological fluids) [74]. Several parameters, such as the cross-linking degree of the polymer and its hydrophilicity [75,76,77], can significantly affect their properties. Hydrophilic polymers show the ability to swell in water and to hold more than 10% water within the gel’s network. This property depends on the presence of different functional groups on the polymeric chain, such as carboxylic (-COOH), hydroxylic (-OH), amidic (-CONH), and sulphonic (-SO_3_H) ones [78]. Hydrogel texture can be influenced by modifications in the structure and functionality of the polymer, in changes of its concentration and in the use of different cross-linkers. Moreover, new hydrogels have been studied and realized in different fields of engineering (environmental, biomedical), biotechnology, and many other contexts [79]. The growing interest in the topic can be easily checked by a quick search for the term “hydrogel” in the PubMed database that shows a significant exponential trend in the number of published papers regarding this item (Figure 3).

The first hydrogels reported in the literature were described by Wichterle and Lím [80] who used poly (hydroxyethyl methacrylate) (PHEMA) hydrogel for soft disposable contact lenses. There are several advantages of a hydrogel in such an application: they are elastic, biocompatible, maintain the natural eye humidity, and allow oxygen diffusion from the outside.

Hydrogels can be ranked as natural and synthetic according to the nature of their polymers, configuration, electrical network charge, crosslinking, and physical appearance. Natural hydrogels such as proteins and polysaccharides have recently been replaced by synthetic polymers, due to the great advantages regarding, for example, biocompatibility and strength [81]. Synthetic hydrogels are obtained starting from homopolymers or copolymers by several preparation techniques such as bulk, solution, and suspension, by chemical or physical cross-linking pathways [82,83,84,85]. The “three-dimensional polymerization” occurs starting from a hydrophilic monomer with a crosslinking agent by direct or indirect crosslinking. Chemically cross-linked hydrogels are the most favorable since they have a good mechanical strength. They present covalent junctions between the polymeric chains, added by the cross-linking method [86]. In addition, the polymerization can be facilitated by employing specific initiators (ammonium peroxodisulphate, benzoyl peroxide or 2,2-azo-isobutyronitrile) or by UV and gamma radiations with electron beam. Another technique is presented by suspension polymerization or inverse-suspension polymerization which consists of dispersing a monomer in a hydrocarbon phase to give a W/O process with the addition of a suspending agent with a low hydrophilic–lipophilic balance (HLB) [87].

The most significant properties of hydrogels regard swelling, mechanical and rheological properties, biodegradability, and biocompatibility. The phenomenon of hydrogel swelling is the behavior that is observed when, in deep contact with water, the polymeric material relaxes its network system and expands towards a certain state of solvation [88].

The most important factors affecting the swelling properties of hydrogels are represented by the nature of solvents, the solvent–polymer interaction parameters and the network density [89]. Several studies regarding swelling have been carried on by immersion of the dried hydrogel into water and subsequently by removing and weighing it (after drying the medium excess from the surface). For the percentage of swelling ratio, the Rs of hydrogels can be defined by Equation (1):

(1) Rs=Ws−Wd/Wd × 100

where *Ws* is the weight of the swollen hydrogel and *Wd* is the original weight of the hydrogel before immersion in water. The *Rs* values were dramatically affected by the crosslinking degree: increasing this parameter decreases the *Rs* value, while with a low cross-linking degree, a higher hydrodynamic free volume of the network is observed because it has to store a greater amount of water, increasing the matrix swelling. The mobility and relaxation of the polymeric chains are prevented by an increase of the cross-linking degree, which prevent water mobility and consequently decrease the *Rs* values [90]. Water retention, *Wr*, can be obtained from Equation (2):

(2) Wr=Wt−Wd/ Ws × 100

where *Wt* represents the complexive mass of the hydrogels, at a defined time interval, *Ws* and *Wd* represent the hydrogel weight in the swollen and dried state, respectively [91]. Another theory that explains the swelling behavior of a hydrogel is the one proposed by Flory–Rehner, using Gibbs free energy, about equilibrium swelling theory [92]. This theory is based on the following equation:

(3) ΔG total= ΔGmix+ ΔGel

where *ΔG total* represents the complexive free energy of the polymeric network, *ΔGmix* represents the free energy contributions deriving from the enthalpy of mixing, and *ΔGel* represents the free energy contribution derived from the elastic retractile network forces [93].

**Figure 4:**
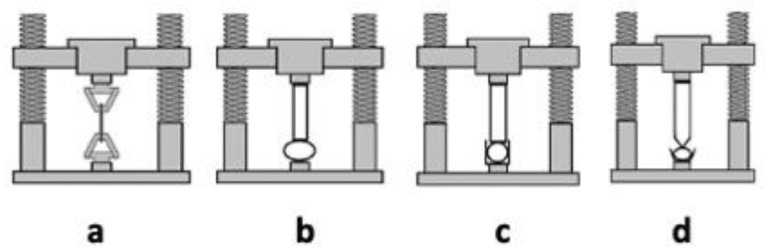
Testing methods applied to assess mechanical properties of hydrogels: tensile testing (**a**), unconfined compression testing (**b**), confined compression test (**c**), and indentation testing (**d**).

Hydrogels present mechanical properties that can be considered significant parameters for several biomedical applications in particular in drug delivery and tissue engineering [91]. A hydrogel should preserve its texture, for a given time, in order to deliver a drug at a required target; this behavior can be affected by the type and concentration of the crosslinking agent. The crosslinking degree ensures the hydrogels’ stable mechanical and elastic properties: an optimal crosslinking degree must be obtained to have a relatively strong and yet elastic hydrogel [94]; an increase of this value leads to a stronger hydrogel, even if the higher crosslinking degree decreases the percentage of hydrogel elongation, creating a more brittle structure. Different techniques, such as tension, compression (either confined or unconfined), and indentation testing (Figure 4) can be applied to measure the mechanical properties of hydrogels. During the tensile test (**a**), the sample is placed between two clamps; the two ends, thus secured, are detached by applying a force until breaking [95]. The tensile test takes advantage of a dynamometer with a load cell, obtaining stress-strain curves useful to obtain several mechanical properties (i.e., Young’s modulus, yield strength, and ultimate tensile strength).

The compression tests are carried out in an unconfined model (**b**) or confined one (**c** and **d**). In the first model (**b**), two plates compress the hydrogel, which is placed between the two punches. In the second test (confined compression, **c**), the hydrogel is confined inside a sample holder and compressed by an upper punch. In the indentation testing (**d**), the hydrogels are serrated by a probe, of defined shape, that penetrates the thickness for a given depression, then measuring the specific force needed to lead to this indentation [96].

Rheology is useful to investigate different mechanical properties such as the mechanical strength and flow of hydrogels and can therefore be considered a basic tool for the characterization of industrially significant properties. Moreover, rheological measurements can provide information related to the internal structure of soft materials, according to their response as regards dynamic behavior. They are useful tools for studying bulk phase transitions, in particular solution-to-gel (solgel) transitions, which can be induced by significant changes of pH, concentration, and temperature [97]. The physical structure and rheological properties are significant parameters to be considered for strategic hydrogel applications in biomedical contexts; in this case, the rheological behavior of the studied material is measured by a rheometer whose several available shapes can ensure well-defined conditions of flow for a rheological experiment. “Concentric cylinders” (Couette), “cone-and-plate”, and “parallel disks” are the most commonly applied instruments [98]. Rheology techniques can also be applied for characterizing gelation behavior such as the crosslinking degree and structural properties (homogeneity/heterogeneity) [99]. In the case of hydrogels used for hand sanitization, it is very important to have the correct viscosity that allows the formulation to be dispensed in the appropriate dose and a good spreading coefficient that guarantees the complete covering of the skin. A reasonably high viscosity is relevant for the spreadability of skin formulations. However, it is still not well defined how increasing viscosities from fluid to semi-solid formulations will affect skin penetration. As regards the impact of rheological formulation properties on skin penetration, scientific conclusions are controversial. A recent work reported that the optimal viscosity values for a good hand sanitizer gel are 47,000 to 150,000 mPa.s [100], meeting the standards set by Zatz and Kushla [101].

## 5. Natural and Synthetic Polymers in Sanitizing Hydrogels

A great variety of natural and synthetic polymeric compounds, commonly used in the pharmaceutical, cosmetic and food fields, are available to obtain hydroalcoholic and non-alcoholic sanitizing hydrogels. Each of them is available in several grades, presenting different thickening behavior, rheological properties, solubility, and classification (pharmaceutical, cosmetic, or food grade). Thickening properties can change according to several parameters such as pH, presence of electrolytes, and the addition of excipients. In order to provide a sort of general guide for selecting thickeners in the development of hydrogel hand sanitizers, in Table 3, we have reported the most common synthetic and natural polymers available on the market useful as gelling agents in AFR and ABR, accompanied by the most significant data that can influence their rheological behavior (as reported by suppliers) such as dosage range, eventual maximum alcoholic amount (in the case of ABR) and pH range. As regards “electrolyte tolerance”, we could only give approximative levels (low, good, and very good) as found in several technical data sheets collected, and it must be said that despite the same term, the numerical meaning can be very different from one company to another.

The performance of the classes of polymers reported in Table 3 is well-known as regards aqueous media, but their behavior in hydroalcoholic solvents has not yet been deeply investigated. For this reason, and with the aim of giving useful indications for increased ABR production and development, especially in this pandemic emergency, in Table 4, we have reported a collection of examples regarding alcohol-based hydrogel formulations. The polymer and alcoholic amount and eventual addition of excipients are reported as suggested in the suppliers’ brochures, accompanied by the most meaningful data related to the obtained hydrogel in terms of viscosity and transparency.

### 5.1. Carbomers

Carbomers represent a series of polymers widely used in cosmetic and pharmaceutical products as rheological modifiers. They are cross-linked polyacrylic acid polymers with high molecular weight, show a very efficient thickening capability, and are considered powerful stabilizers at low concentrations in water and hydroalcoholic solutions (0.1 to 3% *w*/*w*). The most common classification groups them according to the cross-linker type: carbomer homopolymers (acrylic acid crosslinked with allyl pentaerythritol or allyl sucrose), carbomer copolymers (acrylic acid and C10-C30 alkyl acrylate crosslinked with allyl pentaerythritol), and carbomer interpolymers (homopolymeric or copolymeric carbomer containing a block copolymer of polyethylene glycol and a long chain alkyl acid ester) [73]. According to the cross-linking density (low, medium, or high) polymers with a specific ability of increasing the viscosity of aqueous systems are provided. Being acidic in their undissociated state, they need to be neutralized with a specific basic organic or inorganic compound to perform as thickening agents. Despite the large number of neutralizing agents useful for aqueous dispersions (such as sodium, ammonium, and potassium hydroxides, aminomethyl propanol, tetrahydroxypropyl ethylenediamine, triethanolamine, diisopropanolamine, and triisopropanolamine), when carbomers are used for hydroalcoholic hydrogels, the neutralizer has to be carefully chosen in order to prevent the polymer precipitation. The most common organic and inorganic bases are the following:

- Inorganic bases, such as NaOH and KOH, specifically for hydro-alcoholic mixtures with a max content of 20% ethanol.
- Triethanolamine is the most suitable neutralizing agent for formulations containing up to 50 to 60% ethanol.

Taking into account the ethanolic amount in ABR (60 to 95%), suppliers and productors of carbomers recommend specific neutralizers, in particular tetrahydroxypropyl ethylenediamine, aminomethyl propanol, and triisopropanolamine [102]. All carbomers can thicken hydroalcoholic systems, but several grades can offer different advantages in terms of aspect and performance, such as higher transparency, better efficiency, and ease of handling also leading to the optimization of the overall aesthetic characteristics of commercial hand sanitizing gels [103,104]. Carbomers have better thickening properties than cellulose derivatives, but the rheological behavior of carbomers in aqueous and hydroalcoholic media shows a reduction of hydrogel consistency in the presence of ethanol, in particular at a polymer concentration of 0.1% *w*/*w* and at low pH values (pH = 4) [105] (Table 3 and Table 4).

### 5.2. Cellulose Derivatives

#### 5.2.1. Hydroxyethyl Cellulose (HEC)

Hyroxyethyl cellulose is a non-ionic partially substituted poly(hydroxyethyl) ether of cellulose. It can be prepared by the reaction of cellulose with ethylene oxide under controlled and basic conditions with sodium hydroxide. The average number of ethylene oxide groups, attached to each glucose residue, is represented by the total molar substitution (MS), while the number of hydroxyl groups for every reacted glucose residue is represented by the degree of substitution (DS). Hydroxyethyl cellulose with DS = 1.5 and MS = 2.5 can be available with different molecular weight grades, corresponding to a different viscosity in aqueous media. L, M, H, and HH refers to low, medium, high, and very high viscosity, respectively. HEC can be dissolved in cold and hot water and it is not soluble in organic solvents. Hydroxyethyl cellulose of type L and M are very soluble in glycerin and present a good solubility in alcoholic solutions up to 60% ethanol [106]. Hydroxyethyl cellulose is not recommended to obtain gel formulations containing > 65% alcohol, because of the low solubility of this cellulose derivative and the turbid aspect (Table 3 and Table 4).

#### 5.2.2. Sodium Carboxymethyl Cellulose (CMC)

CMC is an anionic rheological modifier, soluble in water at any temperature and giving clear colloidal systems at 1 to 6% [107]. It is available in different useful types according to DS and MS and it is classified by the letters “F” for food, “CS” for cosmetic, and “PH” for pharmaceutical use, according to American (USP), European (Ph. Eur), and Japanese (JP) pharmacopoeia [108,109,110]. Even though it is not soluble in a large number of organic solvents such as ethanol (95%), CMC is able to provide transparent systems in alcoholic solutions up to 40% ethanol. In higher amounts (up to 60% ethanol), it is possible to disperse CMC but obtain turbid systems. According to the literature, CMC is not useful for the preparation of hand sanitizers, being useful only for obtaining gels with ethanol up to 50% (Table 3).

#### 5.2.3. Hydroxypropyl Methylcellulose (HPMC)

HPMC is a cellulose ether derivative widely applied in pharmaceutical formulations, and cosmetic and food products. The numeric code in the nomenclature indicates different types of HPMC, related to different percentages of methyl and hydroxypropyl groups and molecular weights [111,112]. HPMC is a widespread thickener for aqueous solutions and for a great number of binary solvent systems. Moreover, 2% HPMC (especially HPMC 2910) has a good solubility at high percentages of ethanol and isopropanol in water, allowing to obtain transparent gels with an appropriate viscosity [113] (Table 3 and Table 4).

## 6. Other Excipients in Hand Sanitizers

An important side effect in the use of hand rubs is skin dryness, due to over frequent application. Hydrating, refatting, and emollient agents can protect from the excessive drying effect of alcohol and detergents [114,115,116]. Glycerin is the most widespread humectant in sanitizing hand rubs [66]; in order to maintain the antimicrobial activity, the recommended concentration is 0.50 to 0.73%, because it still offers the necessary skin protection [117,118]. Glycerin is able to reduce the antimicrobial activity of several ABRs [119] if used at a concentration of 1.45% (*v*/*v*), and an excessively high concentration can extend the drying time of the hand rub, increasing the sticky sensation on the hands. Other emollients can be used to improve skin tolerance and consumer acceptance. Propylene glycol can be used at concentrations of 2 to 5%; ethylhexyl glycerin, dexpanthenol, and fatty alcohols can be added without decreasing antimicrobial efficacy [120]. Among several hydrating ingredients, Aloe vera gel has also been used in several cosmetic handrubs, increasing consumer interest as it is considered natural. It can be used in combination with glycerin or propylene glycol and can contribute to the firmness of gel formulations if used at very high concentrations.

## 7. International Handrub Sanitizers Regulation

### 7.1. US Regulation

In the USA, sanitizing handrubs are considered over-the-counter (OTC) drugs that must comply with the requirements set out in the 1974 monograph for hand sanitizers. However, more recently, due to the COVID-19 emergency, the U.S. Food and Drug Administration (FDA) has liberalized the preparation of alcohol-based hand sanitizers as long as the registered compounder follows the published formulas and guidelines for industry, allowing preparation and selling of these sanitizing products for the pandemic emergency. This accord was published on March 20, 2020 under the title, “Policy for Temporary Compounding of Certain Alcohol-Based Hand Sanitizer Products.” [6]. The products do not require registration provided; they do not deviate from the provisions set up by the FDA for that product category. An alcohol hand sanitizer needs to contain ethyl alcohol or ethanol at a level of 60 to 95% or isopropyl alcohol at a level of 70 to 91.3%. It must also be made under GMP (Good Manufacturing Practices) requirements, and the production facility must be listed with the FDA.

### 7.2. Europe Legal Framework

Alcohol-based gels marketed in Europe can follow two different regulations: Cosmetic Products Regulation (Regulation (EU) N° 1223/2009, 2009) or Biocidal Products Regulation (Regulation (EU) N° 528/2012, 2012). With the presence of an active substance, the main purpose of the product and the label claims determine the choice of the regulation market. A sanitizing hand gel can be considered as a cosmetic product if it does not make any biocidal claims. In this case, a Cosmetic Product Safety Report (PCSR), label review, Product Information File (PIF), Cosmetic Product Notification Portal (CPNP), and Responsible Person (RP) are needed [121]. A product containing an active substance, with a biocidal purpose, with biocidal claims on the packaging and in advertising falls within the scope of the Biocidal Products Regulation, being considered as Product Type 1 (PT1)—human hygiene biocidal product. The BPR provides alternative provisions to standard requirements (Article 55) allowing member states to fast-track biocidal products when there is a public health emergency. In the context of the COVID-19 emergency, to lighten the difficulties of companies and member state authorities, the European Commission has also issued an important guideline in order to assist in the production of hand cleaners and sanitizers and in the application of Regulation (EU) 1223/2009 and Regulation (EU) 528/2012) [122].

## 8. Conclusions and Perspectives

The request for hand sanitizers is increasing due to the pandemic emergency, and the use of instant hand rub gel sanitizers is becoming more and more popular thanks to their fast action and good performance in killing microorganisms. In this review, the authors tried to report the state-of-the-art, current knowledge and trends about hand sanitizers, focusing their attention on the importance of hydrogels to obtain efficient formulations, easy to use, fast in delivery and action and not too aggressive for the skin. In this regard, we have also collected and reported a detailed list of the most commonly used polymeric thickeners, accompanied by their principal commercial trade names and behavior in solution, as a potential guide for the formulation design and production of quality sanitizing hydrogel hand rubs. The aim was also to give alternative solutions to the use of carbomers which seems to be the best performing rheological modifier for gel formulations especially as regards viscosity, electrolyte tolerance, and aspect (Clarity—transparency), but whose lack among suppliers (especially during the pandemic) has created troubles and bad formulative solutions in the past months. Once the polymer of interest has been identified, it is very important to select the correct amount of alcohol and antiseptic agent, in conjunction with other compatible excipients, in order to obtain performing formulations, easy to use and in accordance with the current legislation.

## Figures and Tables

**Table 1:** Liquid formulations recommended by the WHO.

**1** Ethanol 96%: 80% *v*/*v* Hydrogen peroxide 3%: 1.45% *v*/*v* Glycerol 98%: 0.125% *v*/*v* Water
**2** Isopropyl alcohol 99.8%: 75% *v*/*v* Hydrogen peroxide 3%: 1.45% *v*/*v* Glycerol 98%: 0.125% *v*/*v* Water

**Table 2:** Most common antiseptic agents used in AFR, with their suggested active percentage in sanitizing handrubs and mechanism of antimicrobial action [57].

Antiseptic Compounds	Suggested % Amount	Chemical Agents	Antimicrobial Activity
Quaternary ammonium compounds	≤1%	Benzalkonium chloride, benzethonium chloride, cetrimide, cetylpyridium chloride	Lower surface tension. Enzyme inactivation. Degradation of cell proteins.
Iodine/Iodophors	≤1%	Povidone-iodine (polyvinylpyrrolidone with iodine)	Penetration through the cell membranes, subsequent cell inactivation due to the formation of complexes with amino acids and unsaturated fatty acids. Subsequent impaired protein synthesis and alteration of cell membranes.
Chlorine derivatives	3–6%	Chloroxylenol (phenolic compound)	Inactivation of bacterial enzymes. Alteration of cell walls.
		Chlorhexidine (bisbiguanide)	Disruption of cytoplasmic membranes.
		Triclosan	Penetrate cytoplasmic bilayer.
		Sodium hypochlorite	Oxidation of cell proteins. Oxidation of DNA and/or RNA.

**Table 3:** Most common natural and synthetic polymers useful as rheologic modifiers in hydroalcoholic and non-alcoholic sanitizing hydrogels.

Chemical Name (INCI)	Trade Name (Supplier)	Dosage Range (%)	Max EtOH Amount (% *v*/*v*)	pH Range	Electrolyte Tolerance
Carbomer	CARBOPOL ULTREZ 10 (Lubrizol)	0.1 to 0.5	60 to 95 (according to neutralizer)	5 to 9	low
	CARBOPOL 980 (Lubrizol) ASHLAND 980 Carbomer (Ashland)	0.1 to 0.5	60 to 80 (according to neutralizer)	5 to 10	low
	TEGO Carbomer 140 (Evonik)	0.05 to 1.0	60 to 95	3 to 10	low
	CARBOPOL 940 (Lubrizol) ASHLAND 940 Carbomer (Ashland)	0.1 to 0.5	60 to 95 (according to neutralizer)	5 to 10	low
Acrylates / C10–30 Alkyl Acrylate Crosspolymer	CARBOPOL ULTREZ 21 (Lubrizol)	0.1 to 0.5	60 to 95 (according to neutralizer	5 to 10	low
	CARBOPOL ULTREZ 20	0.1 to 0.6	60 to 95 (according to neutralizer)	4 to 11 (lower viscosity)	low
	TEGO^®^ Carbomer 341ER (Evonik)	0.05 to 1.0	60 to 95	4 to 11 (lower viscosity)	low
Cellulose gum (CMC)	AQUALON (BLANOSE) (Ashland)	1.0 to 2.0	60	3 to 12	low
Hydroxyethylcellulose (HEC)	NATROSOL 250 HHR CS (Ashland)	0.2 to 2.5	65	3 to 12	good
	TYLOSE HS (Shin-Etsu)	0.5 to 2.0	62	3 to 12	good
Hdroxypropylmethyl cellulose (HPMC)	BENECEL E10M (Ashland) TYLOPURE DG (Shin-Etsu)	0.2 to 2.0	70	5 to 8	good
Hydroxypropyl Guar	JAGUAR HP 120COS (Solvay)	1 to 1.5	70	4 to 8	very good
Ammonium Acryloyl dimethyltaurate/ Beheneth-25 Methacrylate Crosspolymer (pre-neutralized)	ARISTOFLEX HMB (Clariant)	0.5 to 1.0	70	2.5 to 8	low
Ammonium Acryloyl dimethyltaurate/VP Copoymer (Pre neutralized)	ARISTOFLEX AVC (Clariant)	0.5 to 1.0	70	4 to 8	low
Sodium Acryloyldimethyltaurate/ VP Crosspolymer (Pre neutralized)	ARISTOFLEX AVS (Clariant)	0.5 to 1.2	70	4 to 11	low
Polyacrylates Crosspolymer-11 (pre-neutralized)	ARISTOFLEX VELVET (Clariant)	0.5 to 1.5	70	3 to 8	low
Sodium Polyacryloyl dimethyltaurate	ARISTOFLEX SILK (Clariant)	1 to 1.5	70	2 to 11	good
Polyacrylamide—C_13–14_-isoparaffin—laureth 7 (Pre-neutralized)	SEPIGEL 305 (Seppic)	0.5 to 5.0	70	3 to 12	very low
Polyacrylate 13—polyisobutene—polysorbate 20 (Pre-neutralized)	SEPIPLUS 400 (Seppic)	0.1 to 2.2	65	3 to 12	good
Hydroxyethyl acrylate—sodium acryloyldimethyl taurate copolymer (Pre-neutralized)	SEPINOV EMT10 (Seppic)	0.5 to 3.0	65	3 to 12	good
Polyacrylate crosspolymer—6 (Pre-neutralized)	SEPIMAX ZEN (Seppic)	0.8 to 2.0	70	2 to 8	very good

**Table 4:** Hydrogel ABRs: most common commercial polymers, corresponding polymeric dose, suggested ethanolic percentage, clarity and viscosity of the obtained hydrogel are reported.

Polymer Trade Name	Polymer Amount (%)	EtOH Amount (% *v*/*v*)	Notes (Additives)	Hydrogel Aspect *	Hydrogel Viscosity (mPa.s) **
CARBOPOL ULTREZ 10 (Lubrizol)	0.5	70	0.35% aminomethyl propanol (neutralizer)	Clear	3500 to 4500
ASHLAND 980 Carbomer (Ashland)	0.35	73	0.15% aminomethyl propanol (neutralizer) 1.5% glycerin	Clear	15,000 to 25,000
TEGO^®^ Carbomer 341 ER (Evonik)	0.3	70	0.5% tetrahydroxy propyl Ethylenediamine, (neutralizer) 3% glycerin	Clear	4350
CARBOPOL 940 (Lubrizol)	0.5	50	triethanolamine up to pH 6	Clear	1200
CARBOPOL ULTREZ 21 (Lubrizol)	0.2	60	0.25%Triisopropanolamine (neutralizer) 0.5% propylen glycol	Clear	8000 to 12,000
CARBOPOL ULTREZ 20 (Lubrizol)	0.2	60	0.25%Triisopropanolamine (neutralizer) 0.5% propylen glycol	Clear	4000 to 6000
NATROSOL 250 HHR CS (Ashland)	1.4	65	-	Turbid	14,700
Tylose HS 100000 (Shin-Etsu)	1.5	62	triethanolamine up to pH 8.5 2% glycerin	Turbid	37,000
Benecel E10M (Ashland)	1.5	75 65	1.5% glycerin 2.0 % glycerin	Clear	4000 to 6000 1325
TYLOPURE DG 4T (Shin-Etsu)	2.0	65 75 85	3.0 % glycerin	Clear	7768 6184 5352
JAGUAR^®^ HP 120 COS (Solvay)	1.2	75	citric acid (pH adjuster)	Clear	3500 to 5000
ARISTOFLEX^®^ HMB (Clariant)	1.0	62	-	Clear	20,000
ARISTOFLEX^®^ AVC (Clariant)	1.0	65 75	-	Clear	30,000 40,000
ARISTOFLEX^®^ VELVET (Clariant)	0.45 to 0.5	70 to 80	2% glycerin	Clear	2940 to 2100
ARISTOFLEX^®^ SILK (Clariant)	1%	60	1.5% glycerin	Clear	14,000
SEPIGEL 305 (Seppic)	1.6	65	3% glycerin	Turbid	8000
	2.0	70	0.2% sepimax zen	Turbid	8000
	2.2	65	1% SIMULSOL 1293 (solubilizing nonionic Surfactant—Seppic)	Clear	7148
	3	65	-	Turbid	35,000
SEPIPLUS 400 (Seppic)	2.25	65	-	Turbid	46,000
SEPINOV EMT10 (Seppic)	0.80	65	sprayable	Turbid	580
	1.50	65	-	Turbid	8300
SEPIMAX ZEN (Seppic)	0.80	66	3% glycerin	Clear	8900

* Clear = % Transmission at 420 nm over 90%. ** Viscosity was measured with Brookfield viscometer at 25 °C.
